# Analysis of the Parameters Affecting the Stiffness of Short Sisal Fiber Biocomposites Manufactured by Compression-Molding

**DOI:** 10.3390/polym14010154

**Published:** 2021-12-31

**Authors:** Antonio Pantano, Carmelo Militello, Francesco Bongiorno, Bernardo Zuccarello

**Affiliations:** Dipartimento di Ingegneria, Università degli Studi di Palermo, 90128 Palermo, Italy; carmelo.militello01@unipa.it (C.M.); francesco.bongiorno01@unipa.it (F.B.); bernardo.zuccarello@unipa.it (B.Z.)

**Keywords:** biocomposites, natural fibers, sisal, numerical methods

## Abstract

The use of natural fiber-based composites is on the rise in many industries. Thanks to their eco-sustainability, these innovative materials make it possible to adapt the production of components, systems and machines to the increasingly stringent regulations on environmental protection, while at the same time reducing production costs, weight and operating costs. Optimizing the mechanical properties of biocomposites is an important goal of applied research. In this work, using a new numerical approach, the effects of the volume fraction, average length, distribution of orientation and curvature of fibers on the Young’s modulus of a biocomposite reinforced with short natural fibers were studied. Although the proposed approach could be applied to any biocomposite, sisal fibers and an eco-sustainable thermosetting matrix (green epoxy) were considered in both simulations and the associated experimental assessment. The results of the simulations showed the following effects of the aforementioned parameters on Young’s modulus: a linear growth with the volume fraction, nonlinear growth as the length of the fibers increased, a reduction as the average curvature increased and an increase in stiffness in the *x*-*y* plane as the distribution of fiber orientation in the z direction decreased.

## 1. Introduction

The use of biocomposites, i.e., materials generally obtained by reinforcing eco-sustainable or renewable matrices using natural fibers, is increasing significantly in many industrial sectors (automotive, construction, shipbuilding, etc.) [1,2,3]. Thanks to their eco-sustainability, these innovative materials make it possible to adapt the production of components, systems and machines to the increasingly stringent regulations on environmental protection [4,5]. Moreover, the use of natural fibers, e.g., sisal, reduces production costs [6], weight and operating costs. Optimizing the mechanical properties of biocomposites is an important objective of applied research in order to extend their use to both nonstructural and semistructural applications [7].

As for composites reinforced by synthetic fibers, angle-ply laminates made using continuous natural fibers are relatively expensive to produce, whereas those made with chopped fibers randomly distributed are inexpensive and can easily be mass produced. For these reasons, the latter are interesting alternative materials for lightweight components in automotive, naval and civil fields, where the optimization of stiffness is, in general, the main objective. To optimize their adoption, numerical models could be very useful, e.g., to study the effect of the main manufacturing parameters on composite stiffness. Although there are several numerical models for randomly distributed short fibers, the literature on composites characterized by a high-volume fraction (35–40%) of high aspect ratio fibers is limited. Mainly, three methods exist: analytical homogenization methods, classical laminated plate theories and finite element analysis (FEA) of proper representative volume elements (RVEs).

Analytical homogenization methods are usually based on the Eshelby’s strain concentration tensor, and take advantage of the Mori–Tanaka method [8,9,10], which allows the determination of the stiffness matrix of a composite material consisting of randomly arranged inclusions [11,12,13]. However, this method should not be used on composites characterized by a high-volume fraction of fibers; because it is based on a diluted homogenization scheme and particle interactions are not sufficiently accounted for, the Mori–Tanaka approach is accurate only up to a 15–20% volume fraction. Recent works have proposed more advanced homogenization methods for chopped fiber reinforced composites [14,15,16,17,18].

Classical laminated plate theory [19] is computationally efficient but offers no information on the stress state at the micromechanical level.

Thanks to the rapid advance in computation power, the most promising characterization methods for randomly distributed short fiber composites are based on finite element analyses of proper RVEs [20,21,22,23,24,25,26]. A definition of RVE was given by Hill [27], i.e., a portion of the material which is mechanically typical of the composite on average in which enough inclusions are present so that the overall apparent elastic module is effectively independent of the surface tensile and displacement values, as long as these values are macroscopically uniform. It is therefore a statistical representation of the material. The size of the RVE depends on the microstructure of the composite material, and to establish it, different dimensions must be tested by FEA [20,21,22,23,24,25,26].

In a previous numerical and experimental study, the effects of natural fiber curvature on the longitudinal modulus of unidirectional long fiber biocomposites with a green epoxy matrix were analyzed [28].

This work studied, through a proper numerical approach, the effects of average length, curvature, distribution of orientation and volume fraction of the fibers on the main elastic characteristics of a biocomposite reinforced by short natural fibers distributed in a random way. A micromechanical numerical model was developed, which requires the spatial realization of a representative volume element of the composite, capable of accurately reproducing the actual characteristics of the fibers and biocomposites. The originality of this research mostly lies in the development of an advanced modelling technique which is able to perform very accurate simulations of biocomposites reinforced by short natural fibers, and in particular, the ability of the model to deal with very high volume fractions of fibers. Agave sisalana fibers, which are used for the reinforcement of biocomposites in the automotive sector, were considered; their peculiar characteristics are described in the literature through specific tensile and pull-out tests on single fibers. At present, biocomposites reinforced by vegetable fibers represent a valid, environmentally friendly alternative to glass fibers, as well as to traditional metals (steel and aluminum), thanks to their low density (1.5 g/cm) [29,30]. Particularly interesting is sisal fiber, which is characterized by short renewal times, high availability on the market and the possibility of cultivation in marginal soils, but also by good resistance and stiffness combined with low cost (less than 0.5 €/kg) and good compatibility with many polymeric matrices [29,30,31,32,33]. An intimate fibrillar structure also makes it the natural fiber with the highest hardness, i.e., comparable to that of fiberglass. An eco-sustainable thermosetting matrix (green epoxy) was considered as the matrix.

The model was validated by comparison with the experimental results obtained through a series of experimental tests carried out on a manufactured sisal green epoxy biocomposite produced by optimized compression molding.

## 2. Materials and Methods

### 2.1. Materials

For the realization of the biocomposites used in the present work, the fibers were opportunely extracted from mature leaves of agave sisalana. The selection process of the structural fibers, already optimized in previous works [29,30,31,32,33], consisted of selecting only the perimeter fibers from the middle third of mature leaves, i.e., 4–5 years old, discarding the less resistant nonstructural central fibers; see [32] for more details. In order to maintain a high degree of renewability, the fibers were only subjected to a manual cleaning process and subsequent drying, without any surface treatment with caustic soda or other detergent.

In brief, the selected fibers had a typical horseshoe transversal section with a mean diameter in the range of 100 to 250 μm, i.e., a value that was, in practice, about 10 times greater than that of the common synthetical fibers used in the polymer matrix composites (PMCs). As with all the sisal fibers, they contained characteristic subfibers, having a diameter between 10 and 30 μm, with walls made of hemicellulose and lignin reinforced by cellulose spirals with winding angles of about 20°. Also, their composition was comparable with those typically reported in literature for sisal fibers (40–88% lignin, 8–24% cellulose, 2–28% hemicellulose). The specific weight was about 14.4 kN/m^3^, i.e., significantly lower than that of the synthetical fibers, and the main tensile properties, determined by a single fiber tensile test, were as follows: Young modulus *E_f_* = 37 GPa, tensile strength *σ_f,R_* = 685 MPa and tensile strain *ε_f,R_* = 1.7%.

Once selected, the fresh fibers, without any pretreatment, were cut with an optimal length of 4 ± 2 mm [34] and mixed with a green epoxy matrix produced by the American Entropy Resin Inc. (San Antonio, CA, USA), named SUPERSAP CNR, with IHN -type hardener. Compared to traditional petroleum-based epoxy resins materials, the green epoxy used contained biobased renewable materials originating as coproducts or from waste streams from other industrial processes, such as the production of wood pulp and biofuels. Such an epoxy resin is produced using an ecofriendly manufacturing process through green chemistry, sustainable raw materials and efficient manufacturing conserving energy, minimizing harmful byproducts and reducing greenhouse gas emissions. Through a life cycle assessment (LCA), the producer demonstrated how SUPERSAP CNR significantly reduces the environmental impact of the products [35]. As widely shown in previous studies [36,37] by the same authors, this matrix exhibits an almost linear elastic behavior; the main static mechanical characteristics are as follows: density *ρ_m_* = 1.05 g/cm^3^, tensile strength *σ_m,R_* is about 50 MPa, Young *E_m_* modulus = 2.7 GPa, and tensile stress strain *ε_m,R_* = 4%.

### 2.2. Manufacturing

**Figure 1:**
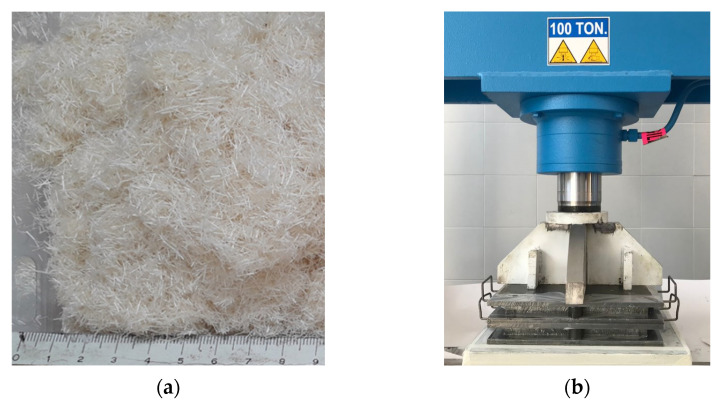
(**a**) Short optimized sisal fibers, (**b**) the double mold and hydraulic press used in the compression-molding operation.

Starting from the 700 ÷ 800 mm long sisal fibers, short sisal fibers of about 4 mm in length were obtained; see Figure 1a. They were first subjected to a drying treatment in a resistance oven with a controlled temperature of 70 °C for 60 min.

The manufacture of the biocomposites was carried out by manually mixing the dry short sisal fibers and the resin in a special container in order to soak all the fibers well, and then distribute them in a special removable mold measuring 260 × 260 mm. In order to obtain biocomposite laminates characterized by low concentrations of voids, and to obtain a final thickness of 3.5 mm with a 35% volume fraction of the fibers, the optimized compression-molding process developed and tested in [29] was carried out using a 100 ton hydraulic press for a duration of about 24 h at a maximum pressure of about 8.3 bar (see Figure 1b). In detail, the initial impregnation of the fibers took place with an excess of resin, and the desired final fiber volume fraction was obtained by suitably adjusting the final thickness of the panel and the weight of the fibers used for each lamination as reinforcement. These factors were determined by the input data, such as lamination surface (mold measuring 260 × 260 mm) and the density of the fibers (1.45 g/cm^3^).

**Figure 2:**
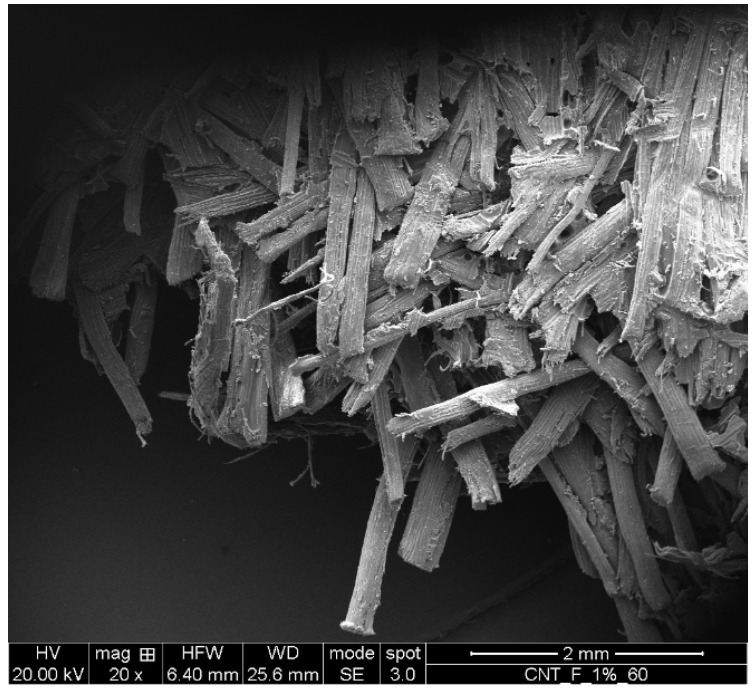
SEM micrographs of the fracture surfaces of the biocomposite subjected to static tensile loading.

Figure 2 shows the SEM micrograph of the fracture surfaces of the biocomposite subjected to static tensile load up to failure. It can be seen that fiber damage was observed only near the fracture surface, i.e., there were no damaged fibers in the remaining areas due to the compression-molding process.

### 2.3. Mechanical Tests

The various biocomposite laminates produced were preliminarily characterized by their tensile strength. An MTS 810 servo-hydraulic test machine with a knife edge extensometer having a 25 mm measuring base was used for the static characterization. The tests were performed in accordance with the ASTM D3039/D standard, in displacement control with an actuator displacement speed of 1 mm/min. Three specimens measuring 25 × 160 mm were tested for each sample. Table 1 shows the experimental results of the tensile tests, in terms of tensile strength, *σ_R_*, and Young’s modulus, *E*, of the biocomposite analyzed along with the same mechanical characteristic of the constituent materials, fibers and matrix.

### 2.4. Methods

Simulations were performed using finite element analyses on representative volume elements. The RVEs, which are extremely complex for high fiber volume fractions, were prepared with the help of the Digimat software [38]. Representative volume elements of various sizes were prepared and analyzed once under periodic boundary conditions (BCs) and once with symmetry BCs applied on three sides parallel to the planes of the chosen coordinate system. A minimum size was identified to guarantee both that the calculated value of the Young’s modulus of the composite was the same in both cases and that it remained constant if the size increased further.

The dimensions used of all RVEs were 5 × 5 × 1.25 mm, which, in the case of *V_f_* = 0.35, indicated presence of about 500 short fibers or portions of short sisal fibers, with an average diameter of 0.2 mm and an average length of 4 mm. Each fiber interacted with the matrix by means of a bonded type relationship, which indicated perfect fiber–matrix adhesion. The meshes ensured convergence of the solutions. On average, the number of quadratic, three-dimensional, tetrahedral elements used was 2.5 million.

**Figure 3:**
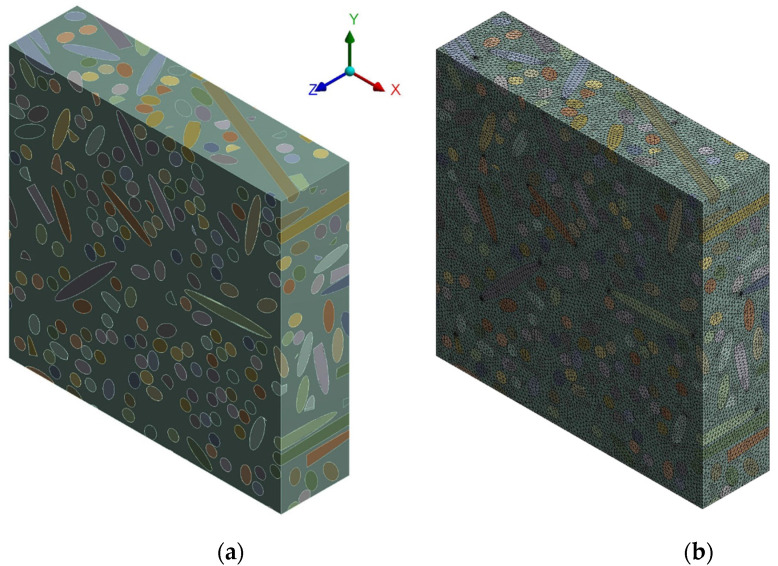
RVE of an epoxy short sisal fiber biocomposite having *Vf* = 0.35: (**a**) 3D view, (**b**) 3D view of the mesh.

Since the numerical model was validated by comparison with the experimental results obtained through a series of tests on biocomposites made with short sisal fibers with *V_f_* = 0.35, the manufacture of which is described in Paragraph 2.2, the RVE had to take into account every feature that characterized the tested samples. In particular, the orientation of the short sisal fibers, which was random at the end of the mixing phase, was subsequently altered by the final manufacturing phase, i.e., compression-molding. During this phase, since the thickness of the specimen was less than the maximum length of the fiber, a given percentage of the fiber lost its random orientation and tended to align itself with the lamination plane (*x*-*y*), resulting in a lower directionality in the direction of compression, i.e., the direction z in Figure 3. This aspect was taken into account by means of an orientation distribution assessment of the RVE of type 0.35 in the *x* direction and 0.35 in the *y* direction, against 0.3 in the *z* direction; see the coordinate system in Figure 3. This specific orientation distribution was determined by exploring the effect of different levels of reductions in the orientation of the fibers in the *z*-axis on the Young’s modulus and comparing the results with the experimental ones.

In DIGIMAT the orientation distribution is defined through an orientation tensor. The orientation tensor is the second order moment of the orientation distribution function, which gives the probability of having a fiber oriented along a given direction; for details refer to [38].

The simulations carried out were all nonlinear, with displacements applied gradually and separately in the *x* and *y* directions, generating a deformation of 3%. The resulting Young’s modulus was the average of the *Ex* and *Ey* results in the two directions, which, for high *V_f_*, differed from each other by less than 1%.

The model was initially validated by comparison with the experimental results previously obtained through a series of tests on biocomposites specially made with short sisal fibers, with *V_f_* = 0.35.

**Figure 4:**
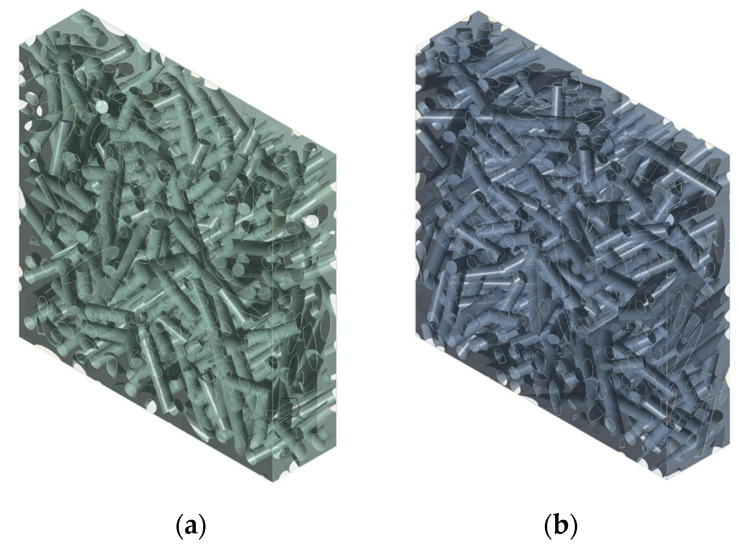
3D transparent view of two different RVEs of an epoxy short sisal fiber biocomposite with *V_f_* = 0.35, as used for the validation of the numerical model: (**a**) first generation run, (**b**) second generation run.

The generation algorithm in DIGIMAT was used to generate two distinct RVEs that shared the same *V_f_* = 0.35 and orientation distribution, i.e., 0.35 in the *x* and *y* directions and 0.3 in the *z* direction; however, these were two very different models, as can be deduced visually from Figure 4, since every generation run produced dissimilar outcomes. The results, in terms of the *E* of the biocomposite, between the two RVEs differed by less than 0.2%; this demonstrated that the model had incorporated a sufficient number of fibers or portions of fibers to guarantee a stable result.

The value of Young’s modulus obtained experimentally for the biocomposite having the same fiber volume fraction, i.e., *V_f_* = 0.35, was *E* = 5.37 ± 0.06 GPa; see Table 1. The numerically calculated Young’s modulus value was 5.5 GPa, i.e., 3% higher than that experimental one, due to the absence of curvature in the fibers. Significant difficulties were encountered when making a RVE characterized simultaneously by *V_f_* = 0.35 and by fibers with nonzero curvature. The influence of the average curvature of the fibers on a RVE with *V_f_* = 0.1 was then analyzed. It was found that even small undulations, i.e., similar to those found in the specimens, caused a reduction in stiffness of between 3% and 5%. Such a result is therefore compatible with the difference between the estimate of Young’s modulus calculated by the numerical model and the experimental value.

Further validation of the numerical approach was achieved by a comparison with the results reported in [29], where, as shown in Figure 16 [29], the experimental values of the Young’s modulus were reported as a function of the volume fraction for the same biocomposite, green epoxy-sisal fibers. The mechanical tests of *V_f_* values between 0.1 and 0.35 showed linear variation of Young’s modulus. This result agrees perfectly with the results of the numerical models presented here, as shown in Section 3.1.

**Figure 5:**
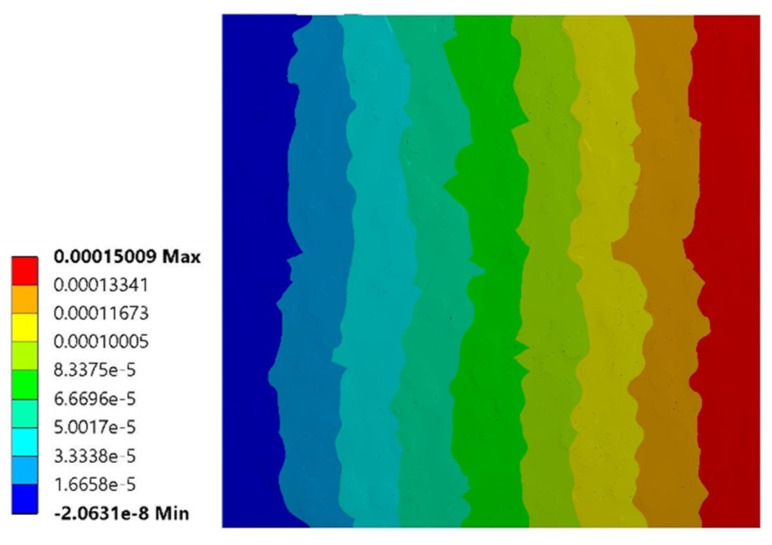
Results of the numerical simulation of simple tensile loading in the *x* direction for an epoxy short sisal fiber composite with *V_f_* = 0.35. Map of displacements in *x*-direction (numerical values represent displacements in meters).

As an example, Figure 5 shows the result of the simulations of one of the two RVEs in terms of displacements in the case of simple tensile loading in the *x* direction.

## 3. Results

### 3.1. Influence of the Fiber Volume Fraction

**Figure 6:**
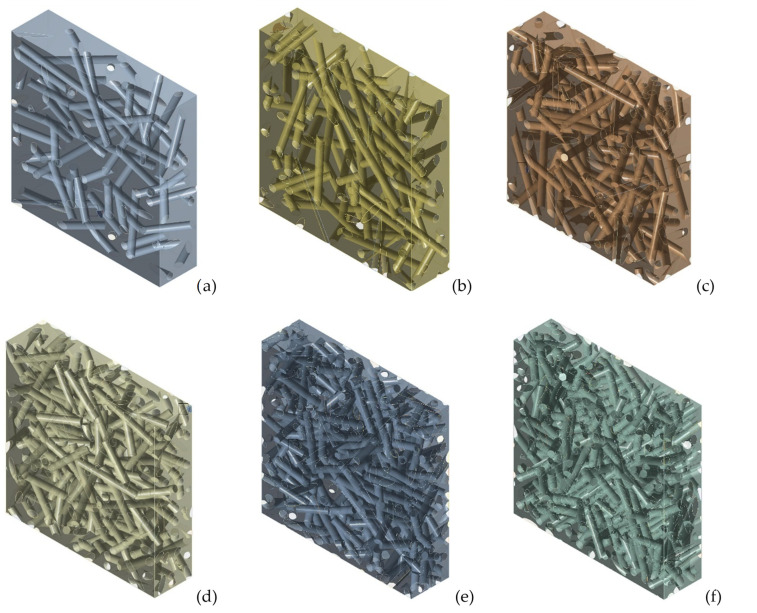
RVE of epoxy-short sisal fiber composites with variable *V_f_*: (**a**) 0.1, (**b**) 0.15, (**c**) 0.2, (**d**) 0.25, (**e**) 0.3, (**f**) 0.35.

One parameter that can significantly influence the stiffness of the composite is the fiber volume fraction, *V_f_*. In order to analyze the influence of this parameter, RVEs were made with the following values: 0.1, 0.15, 0.2, 0.25, 0.3, 0.35. The other parameters were kept constant, i.e., fiber length 4 mm, straight fibers and orientation distribution of type 0.35 in the *x* direction, 0.35 in the *y* direction and 0.3 in the *z* direction. In accordance with [29], a maximum fiber volume fraction of 0.35 corresponds to the maximum tensile strength. As such, higher fiber volume fractions are not used in the practical applications. A 3D transparent view of the six different RVEs is shown in Figure 6.

**Figure 7:**
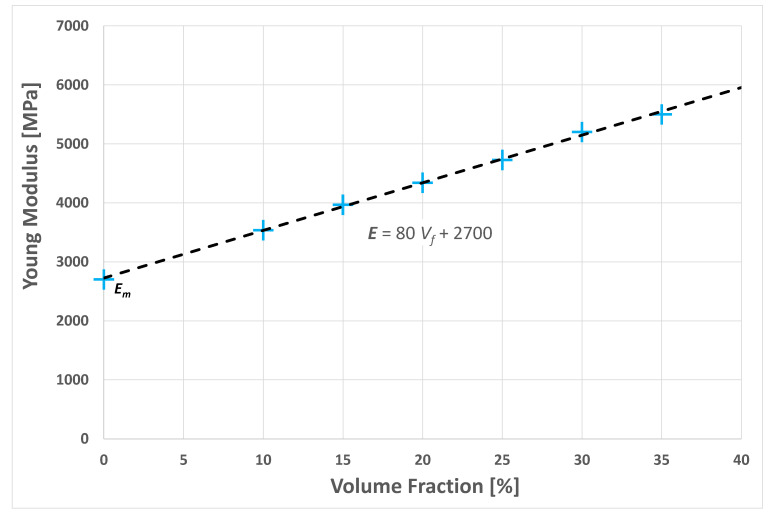
Graph of the Young’s modulus of the biocomposite vs. the fiber volume fraction *V_f_*.

The numerical results showed a fairly linear trend of the *E*(*V_f_*) curve; see the graph in Figure 7.

The interpolation of the numerical results by a linear function yielded a linear relationship:

(1) $$E=E_{m}+kV_{f}$$

where *E_m_* is the Young modulus of matrix *k*, given by:

(2) $$k=\frac{E\left(V_{f}\right)-E_{m}}{V_{f}\%}≅80$$

### 3.2. Influence of the Average Length of the Fibers

**Figure 8:**
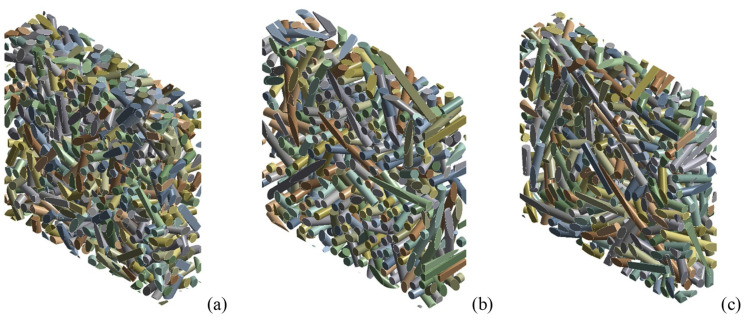
RVE of epoxy resin-short sisal fiber composites with *V_f_* = 0.35 and variable mean fiber length: (**a**) 1 mm, (**b**) 4 mm, (**c**) 8 mm.

As shown by the theoretical model of discontinuous fibers aligned with the composite plane, stiffness and fiber length are significant influencing factors. Stiffness increases in a nonlinear, monotonic way with increasing fiber length [39,40]. Supposing the notion that the fibers always remain linear (i.e., no curvature occurs after mixing with the matrix), and taking into account that, in general, the maximum composite strength corresponds to a length of about 4–6 mm, RVEs were made with average fiber lengths of 1mm, 4 mm and 8 mm. Figure 8 presents 3D views of the fibers with the three RVEs.

**Figure 9:**
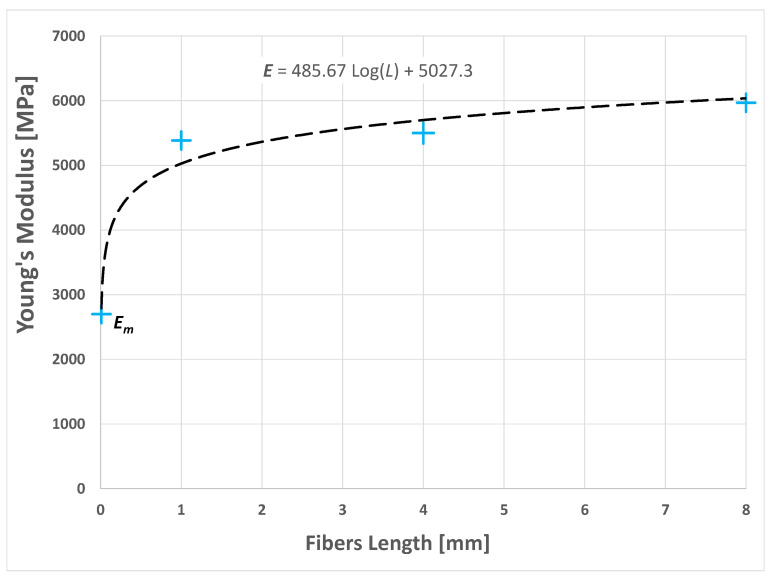
Graph of the resulting Young’s modulus of the composite as the average length of the short sisal fibers varied.

The graph of the numerical results shown in Figure 9 indicates the nonlinear growth of the stiffness of the composite as the length of the fibers increased.

Such results are similar to those of a 2D random fiber characterized by monotonic functions and tending to the well-known long fiber value.

The interpolation of the numerical results by a logarithmic function yielded the following relationship:

(3) E=485.67 Log(L)+5027.3

where *L* is the average length of the fibers.

### 3.3. Influence of Fiber Orientation

**Figure 10:**
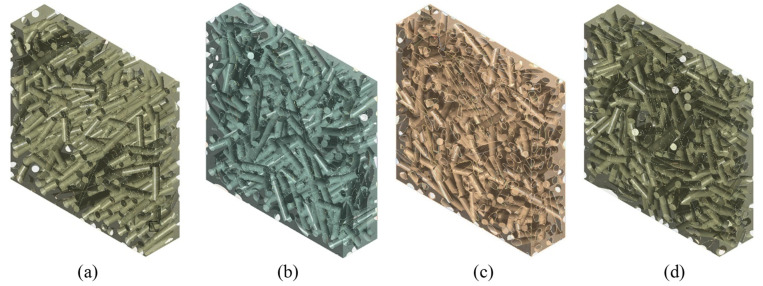
RVE of epoxy resin-short sisal fiber composites with *V_f_* = 0.35 and variable orientation distribution: (**a**) dx = dy = dz = 0.33, (**b**) dx = dy = 0.35 and dz = 0.3, (**c**) dx = dy = 0.375 and dz = 0.25, (**d**) dx = dy = 0.4 and dz = 0.2.

As mentioned above, the stiffness of biocomposites along the x-y plane depends on the fiber distribution on the *z*-axis due to the compression molding along the that axis and to a composite thickness that is less than the maximum fiber length. Considering the optimal *V_f_* value of 0.35, as well as a biocomposites thickness of 3.5 mm and a fiber length of 4 ± 2 mm, the influence of the distribution/orientation of the fibers on the stiffness of the composite in the *x*-*y* plane was analyzed by considering four RVEs with the following fiber orientation distributions: dx = dy = dz = 0.33, dx = dy = 0.35 and dz = 0.3, dx = dy = 0.375 and dz = 0.25, dx = dy = 0.4 and dz = 0.2, where dx, dy and dz represent, respectively, the probability of having a fiber oriented along directions *x*, *y* and *z*. For details, refer to [38]. A 3D transparent image of the four RVEs is shown in Figure 10.

**Figure 11:**
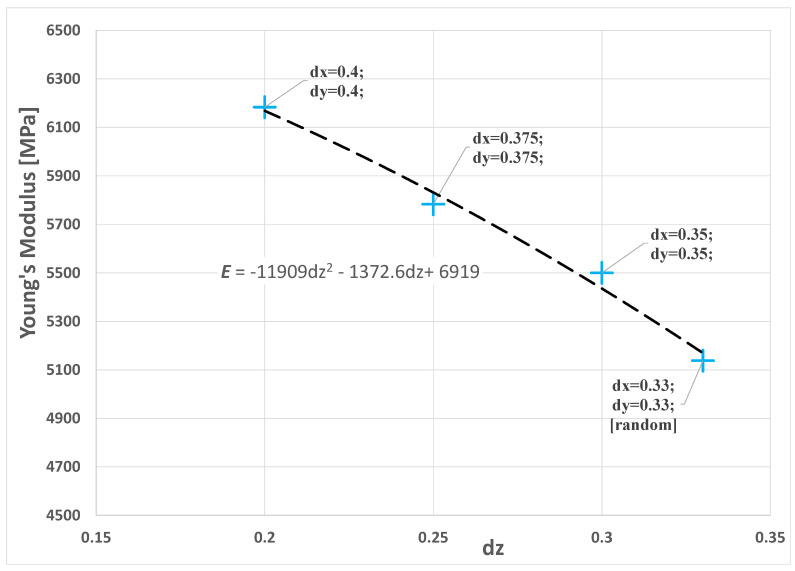
Graph of the resulting Young’s modulus of the composite in the *x*-*y* plane as the distribution of the orientation of the fibers varied.

As expected, the numerical results obtained using such RVEs, and reported in Figure 11, showed a linear increase in the stiffness of the composite in the *x*-*y* plane as the distribution of the fiber orientation along the *z*-axis decreased.

These numerical results are well approximated by the second-degree polynomial below:

(4) $$E=-11909{\;\mathrm{dz}}^{2\;}-1373.6\;\mathrm{dz}+6919$$

### 3.4. Influence of the Curvature of the Fibers

**Figure 12:**
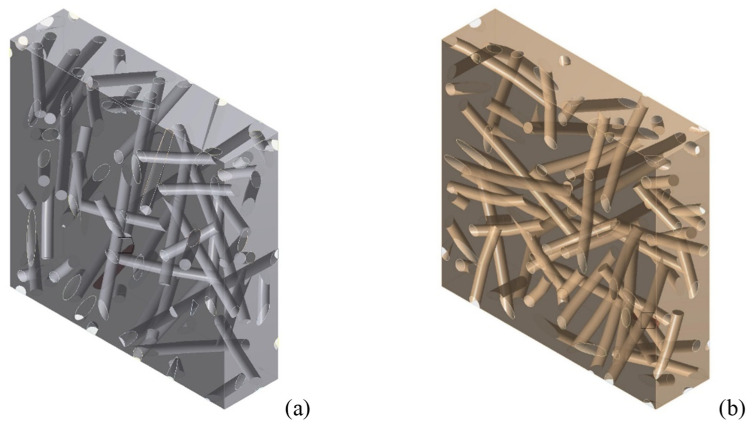
RVE of epoxy resin-short sisal fiber composites with *V_f_* = 0.1 and variable mean fiber curvature: (**a**) 0.75, (**b**) 1.

Since the mechanical mixing of short fibers with the matrix (performed manually or using a mixer) leads to an unavoidable curvature of the fiber due to their limited bending stiffness, it is important to evaluate the appreciable effects of such curvature on the final stiffness of the composite. An analysis of the influence of an average curvature of the fibers on the stiffness of the composite was carried out on an RVE with *V_f_* = 0.1, taking into account the difficulties in generating RVEs characterized simultaneously by large *V_f_* and nonlinear fibers. Further studies are in progress seeking to implement RVE with a higher fiber volume fraction. RVEs were made with the following curvature factors of the fibers: 0.75 and 1.5; see Figure 12. In DIGIMAT, curved inclusions are swept geometries, where the sweep path is a random Bezier curve and the sweep section is a circle. The Bezier curve that was used as sweep path had 11 control points. The first control point was always fixed, while the 10 remaining control points were generated incrementally, with each one being placed at a random distance and orientation relative to the previous one. The curvature factor governed the maximum acceptable change in orientation from one control point to the next; this could range from 0 (which resulted in a straight beam) to 10. This is more a measure of the tortuosity of the fibers than of the actual curvature; for details refer to [38].

As seen in [11,12,13], the numerical results showed a reduction in the stiffness of the composite as the average curvature factor of the fibers increased.

The values obtained from the numerical results may be well approximated by the second-degree polynomial shown below:

(5) $$E=106.67{\;c}^{2}-282.67\;c+3530$$

where c is the curvature factors of the fibers.

**Figure 13:**
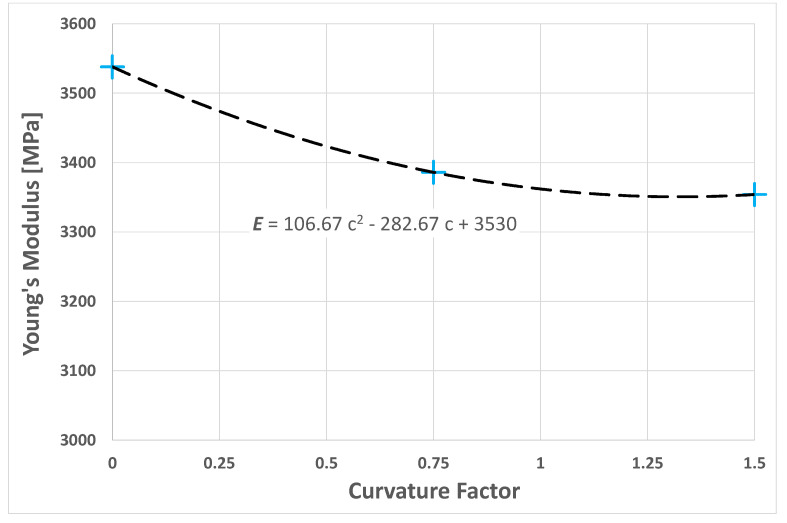
Graph of the Young’s modulus of the composite as the mean curvature factor of the fibers varied in an RVE with *V_f_* = 0.1.

However, the graph in Figure 13 exhibits an apparently asymptotic trend of the Young’s modulus with the curvature factor, which may seem contrary to the expected trend, with an increasingly rapid decrease in stiffness. This was due to the interaction between the fibers which, in the case of a high volume fraction, did not behave as they did when they were surrounded by the matrix alone, but rather, were constrained in their deformation by neighboring fibers.

## 4. Conclusions

The numerical study carried out in this work allowed us to analyze the effects of the volume fraction, average length, curvature and distribution of the fiber orientation on the Young’s modulus of a biocomposite reinforced by natural fibers. In order to assess the numerical results, a biocomposite composed by green epoxy reinforced with sisal fibers was considered. In detail, biocomposite specimens were made by mixing short sisal fibers and resin, and using a compression-molding process to reach a fiber concentration of 35%. A micromechanical numerical model was developed, which required the spatial realization of a representative volume element that was capable of reproducing the actual characteristics of the biocomposites. A comparison with experimental results corroborated the accuracy of the numerical method and the RVEs which were appropriate to carry out the required parametric analyses. The study of the influence of the volume fraction of the fibers showed a linear growth of the Young’s modulus with the volume fraction. The average length of the fibers also had a significant influence on the stiffness of the composite. The results indicated a nonlinear growth in the stiffness of the composite as the length of the fibers increased. An increase in stiffness in the *x*-*y* plane was observed as the distribution of the orientation of fibers in the z direction decreased. Finally, an analysis of the influence of the average curvature factor of the fibers on the stiffness of the composite showed, as was to be expected, a reduction in the stiffness of the composite as the average curvature increased.

## Figures and Tables

**Table 1:** Mechanical properties of materials.

Material	σ*_R_* [MPa]	*E* [GPa]
Sisal fibers	685	37
Green epoxy	50	2.7
Green epoxy-sisal biocomposite with *V_f_* = 0.35	35	5.37

## Data Availability

Data available on request due to restrictions eg privacy or ethical.
